# From ‘Hard’ Neuro-Tools to ‘Soft’ Neuro-Toys? Refocussing the Neuro-Enhancement Debate

**DOI:** 10.1007/s12152-016-9283-6

**Published:** 2016-11-02

**Authors:** Jonna Brenninkmeijer, Hub Zwart

**Affiliations:** 1 Institute for Science, Innovation & Society, Faculty of Science, Radboud University, Heyendaalseweg 135, 6525 AJ Nijmegen, The Netherlands; 2 Theory & History of Psychology, Faculty of Behavioural and Social Sciences, University of Groningen, Grote Kruisstraat 2/1, 9712 TS Groningen, The Netherlands

**Keywords:** Soft neuro-enhancement, Responsible research and innovation, Do-it-yourself technologies, Upstream public engagement, Case study approach

## Abstract

Since the 1990’s, the debate concerning the ethical, legal and societal aspects of ‘neuro-enhancement’ has evolved into a massive discourse, both in the public realm and in the academic arena. This ethical debate, however, tends to repeat the same sets of arguments over and over again. Normative disagreements between transhumanists and bioconservatives on invasive or radical brain stimulators, and uncertainties regarding the use and effectivity of nootropic pharmaceuticals dominate the field. Building on the results of an extensive European project on responsible research and innovation in neuro-enhancement (NERRI), we observe and encourage that the debate is now entering a new and, as we will argue, more realistic and societally relevant stage. This new stage concerns those technologies that enter the market as ostensibly harmless contrivances that consumers may use for self-care or entertainment. We use the examples and arguments of participants in NERRI debates to describe three case studies of such purportedly innocent ‘toys’. Based upon this empirical material, we argue that these ‘soft’ enhancement gadgets are situated somewhere in the boundary zone between the internal and the external, between the intimate and the intrusive, between the familiar and the unfamiliar, between the friendly and the scary and, in Foucauldian terms, between technologies of the self and technologies of control. Therefore, we describe their physiognomy with the help of a term borrowed from Jacques Lacan, namely as “extimate” technologies.

## Introduction

Since the 1990’s, the debate concerning the ethical, legal and societal aspects of ‘neuro-enhancement’ or ‘cognitive enhancement’ (two terms which are often used interchangeably) has evolved into a massive discourse, both in the public realm and in the academic arena. Emerging developments in the neurosciences have raised the possibility that nootropic pharmaceuticals and / or devices, usually developed for therapeutic purposes, may increasingly be used to optimise brain processes in ‘normal’ people who are not impaired by mental illness or cognitive disorders [1]. Countless debates and discussions have been organized, numerous articles have been published and a plethora of opinions have been expressed concerning this issue. And although specific dimensions of human cognitive performance have been highlighted in the course of the debate – genes, neurons, synapses, neurotransmitters, behaviour, etc. – the same set of arguments tends to be raised over and over again. The ethical debate basically revolves around core themes such as authenticity [2, 3], autonomy [4–8], safety and effectivity [9, 10]; competitiveness [11–13], and equity [14–16].

From the very beginning, moreover, there have been two sides in the enhancement debate. Bioconservatives [e.g. 17, 18] argue against it. Their major concern is that enhancement technologies will dehumanize human beings. Neuro-enhancement will make people less authentic, and make society more competitive and less fair. The so-called transhumanists on the other hand [e.g. 19–22] argue in favour of human enhancement technologies. They basically claim that people should be free to decide for themselves and their children, while becoming post- or trans-human should not be seen as a degradation, but rather as an upgrade of human nature and as something which humans have always been striving for anyway (for instance by inventing language, the alphabet, numeral systems, the school system, mechanical instruments, vehicles, etc.).

Besides these normative polarisations, there are also various empirical uncertainties involved. The fact that it is very unclear how many people actually use nootropic substances, for instance, is regularly mentioned [23, 24]. Another point of discussion is how effective neuro-enhancers actually are, or could possibly be [10]. That is, the relevance or even reality of the problem is sometimes questioned [25, 26]. Moreover, if the issue of neuro-enhancement is taken seriously, most people are especially interested in the long-term risks – and since there are no answers to this as yet, the discussion often becomes deadlocked. Perhaps because of this, those people who are either worried or have high expectations about the future of neuro-enhancement often tend to exaggerate the debate. There is a tendency to come up with fairly dramatic (or even fantastic) utopian or dystopian scenarios about humans becoming cyborgs or super-humans, entering a new evolutionary era, while ‘natural’ 1.0 humans will soon become an endangered species [27, 28].

Building on the results of an extensive European project on responsible research and innovation in neuro-enhancement in which we participated (NERRI), we observed that somewhere in between these two more ‘traditional’ pathways – that is, the ‘bioconservative/ transhumanism debate and the effectivity debate - , a less predictable debate on ‘soft’ enhancement technologies seems to unfold as well. We noticed that some people use (or fantasize about) technologies that are not very radical but rather (pretend to be) smooth, controllable and user-friendly. In this article, we would like to emphasize this new tendency and hope to encourage the debate to enter a new and, as we will argue, more realistic and societally relevant stage. In our opinion, the focus of attention should shift from pharmaceuticals such as Ritalin or Modafinil on the one hand and radical cyborgisation of humans on the other to a new generation of optimising devices known as Non-invasive Brain Stimulation devices or NIBS [29, 30], or as Do-It-Yourself brain optimisers [31]. Examples of these technologies are transcranial Direct Current Stimulation (tDCS), transcranial Alternating Current Stimulation (tACS), transcranial Magnetic Stimulation (TMS), and transcranial Focused Ultrasound Stimulation (tFUS). Such brain stimulation technologies are not used for radical self-transformations, but rather function as gadgets that people use to care for themselves or to entertain themselves. This form of enhancement typically takes the form of non-invasive, user-friendly wearable devices. Inspired by this new emerging practice, or even market, of soft (‘smooth’) enhancers, while looking for ways out of the impasse in the established neuro-enhancement debate, we decided to use the empirical material produced in the NERRI project and focus on this quickly evolving arena of non-invasive neuro-enhancement devices.

Most contributions to the academic debate opt for a conceptual and / or normative analysis, assessing and clarifying the various arguments pro and con. Insofar as a more empirical approach is taken, the focus often is on exploring public attitudes towards enhancement [32] or on studying the actual views and experiences of, for instance, student populations [33]. In this paper we will opt for a case study approach focussing on soft enhancement technologies. Three instances of ‘soft enhancement’ will be analysed. We insist on using the quotation marks here because, rather than *being* innocent and harmless, these neuro-toys are *presented* and *promoted* as such. Whether they really are as innocent and playful as they may seem, is one of the issues to be addressed. These gadgets deserve our attention, we will argue, precisely because they are neither trivial nor overly dramatic or futuristic. They are already available, explored and used, at least in an auto-experimental fashion. To arrive at a preliminary assessment of these technologies, we will build on some of the views and arguments which emerged in the course of the NERRI project, in combination with the available literature. We will argue that ‘soft’ enhancement gadgets are situated somewhere in the boundary zone between the internal and the external, between the intimate and the intrusive, between the familiar and the unfamiliar, and between the friendly and the scary. Therefore, we will describe their physiognomy, their technological profile with the help of a term borrowed from Jacques Lacan, namely as “extimate” technologies [34, 35]. Extimate technologies, we argue, are like parasites.^1^ Without us being fully aware of it, they infiltrate our social lives, expose our intimate feelings, and change our ideas of ourselves and each other. And although this relationship is not solely parasitic – since we also enjoy using these small little devices – they come so close to our bodies and beings, we could consider them as rather intrusive.

### Soft Enhancement

NERRI (Neuro-Enhancement: Responsible Research and Innovation^2^) was a mobility and mutual learning project funded by the FP7 Science in Society Initiative of the European Commission, from 2013 to 2016. The aim of NERRI was to contribute to the shaping of a normative framework underpinning the governance of emerging neuro-enhancement technologies. NERRI was conducted by a consortium of 18 partner institutions from 11 European countries.^3^ Each partner collected and analysed relevant literature, conducted interviews with stakeholders, and organized public debates in the form of Mutual Learning Exercises (MLEs). As explained in the NERRI reports, MLEs are events that “aim to bring together various groups of stakeholders (researchers, users, intermediaries, professionals, students, media, broader publics) to facilitate a mutual learning process through mutual exposure of views and experiences, expectations and concerns” [36, p.9, see also 37].

Studying the reports, field notes and some transcripts^4^ of these NERRI events on the one hand clearly revealed the repetitive deliberative trends which are also present in the current academic and public debate mentioned above, namely on the one hand the trend towards trivialisation of the debate (‘How many students take Ritalin before an exam?’; ‘And is it really different from drinking coffee?’) and on the other hand the trend towards over-dramatization of the debate (‘neuro-enhancement techniques will dramatically and irreversible affect human nature’, i.e. the bioconservatism vs. transhumanism debate). Indeed, while the trivialisation trend often stranded in factual uncertainties, the dramatization trend often resulted in endorsing or rejecting the conclusion that we must either save human nature or accept that we are all becoming cyborgs already. On the other hand, and as already indicated in the introduction, a new and (we believe) more interesting area of debate appeared to open up. This debate emerges around this new type of technologies which we already referred to as ‘soft enhancers’ and which purportedly allow users to manage their mood, sleep and level of arousal via measuring, and subsequently influencing, cortical activity in specific regions of the brain

In contrast to technologies that have deep irreversible impacts on neurological health and identity, and which are therefore exclusively applied in therapeutic settings [38, 39], there is already an audience and a market for these ‘softer’ technologies. That is, in contrast to the more scary neuro-tools that are actually inserted into the brain (e.g. Deep Brain Stimulation) or are thought of as having irreversible transformative effects (e.g. brain chips), there is an increasing interest (among early adopters, at least) in neuro ‘toys’: ostensibly harmless contrivances that consumers may use for relatively simple goals, such as paying attention, relaxation (as an alternative to alcohol, soft drugs or smoking), falling asleep, improving one’s gaming skills or learning to play a musical instrument faster.

Neurofeedback is a good example of what we would consider a ‘soft’ technology. This brain computer interface pretends to provide users access to the activity of their ‘brainwaves’, so that they can try to change (for example speed up or slow down) this neural activity. In the context of therapy, neurofeedback is promoted to treat various psychiatric afflictions, from ADHD up to depression, but also physical problems (e.g. motor or bowel problems) and stress-related symptoms (sleeping problems, burn-out), but it is also employed to reach peak performances (boosting concentration or creativity). As a toy it is manufactured in the context of games such as Mindball, in which players influence the movement of a ball across a table with the help of brainwaves, but it also shows up in the context of computer games where it promises to afford users more control over concentration or relaxation. Neurofeedback is widely accepted as a safe tool. Although there is much discussion about its presumed positive effects (such as its therapeutic effect in case of depressions etc.), the side-effects are generally considered to be relatively harmless [e.g. 40–42].

Another technology that is increasingly used as a form of soft enhancement is transcranial direct current stimulation. A tDCS device consists of two electrodes, a low voltage battery and a resistor, which can be used to send very low electric currents to the brain in order to stimulate or inhibit neural activity. tDCS is used as a therapy (for treating ADHD etc.) but also as form of self-optimisation: as a thinking cap to concentrate, or to increase creativity, or as a focus headset to improve gaming performance. In comparison to neurofeedback, however, the general consensus is that in principle there are no serious side-effects, besides skin-irritation or dizziness, but there are warnings that harmful side-effects may occur when the tDCS headset is used inappropriately – for example by mixing up the electrodes or attaching them at the wrong place [43, 44, ].

Technologies such as EEG and tDCS have been discussed on various occasions during NERRI events. Although they were sometimes considered as radical self-transformative and risky, others rather talked or fantasized about these tools as entertaining, caring or life-style gadgets. As indicated, our aim is to distinguish hard (invasive, transformative) from soft (non-invasive, non-transformative) technologies focussing on the latter, but realising that the boundary will be a fluid rather than a rigid one. Nonetheless, our hypothesis is that these relatively new gadgets emerging on the soft side of the distinction will allow us to add relevance and realism to the on-going (academic and public) enhancement debate.

During a workshop on the ethical aspects of tDCS applications,^5^ for instance, participants invented various purposes they would like to use this technology for. They for example fantasized about the possibility to improve their “self-development”. Someone imagined a very small tDCS device that you can ‘stick under your hair, so that nobody notices it, and you can dance all night at a party’. Another participant compared neuro-enhancement with positive psychology, and suggested that we can help children to perform better at school. And yet another participant expressed his desire (his ‘authentic wish’) to learn to play tennis, and that he would like to have a tool that stimulates the learning curve (via brainwaves for instance) to make his wish come true. Other envisioned possibilities were tools to play better mastermind or to make you feel high. Such enabling devices – as they are sometimes called – tend to be seen as relatively innocent and harmless.

In a workshop on the ethical implications of EEG techniques,^6^ the possibility to enhance performances with neurofeedback was discussed. One participant for example mentioned that in several sports, professionals already experiment with this. Helping children with a neurofeedback training to improve concentration was also mentioned, and seen as valuable. Another participant talked about a mobile EEG-device in a backpack – that could monitor brain activity while someone is walking around, which allegedly is in the making to help professional athletes perform better.

We see these examples of soft enhancement as a relatively new form of enhancement which not only seems more realistic compared to some of the thought experiments discussed in the literature, but that may also allow us to open-up the entrenched – and in our opinion somewhat dramatized – debate on transhumanism versus bioconservatism. Soft enhancement technologies are not so much tools to upgrade the brain, but rather toys (gadgets) one can experiment with. Neuro-toys are used to enhance certain skills pertaining to gaming, relaxing, sleeping or paying attention – they are gadgets that allow us to take care of ourselves, or to entertain ourselves. Many of these headsets, apps, watches and other devices are worn close to the body. They can be used for daily practices, such as doing home-work, day-dreaming or meditation.

### Extimate Technologies

With ‘soft’ enhancement technologies, a new type of technological artefacts is entering our lifeworld: small, flexible and easily wearable. These devices are tiny, smooth and subtle, and yet so demanding that they constantly expect us to pay attention. They fit smoothly and imperceptibly into our everyday environments, but precisely because of this smoothness, they may be experienced as pervasive or even uncanny. In other words, they are both intimate and artificial, both alien and familiar, both friendly and intrusive. Therefore, rather than addressing them as “intimate” technologies [45], we will refer to them as “extimate” technologies, building on a concept coined by Lacan [34, 46]. And as indicated, rather than as tools (a term which may suggest hard enhancement), we prefer to refer to them as gadgets or even toys.

These extimate neuro-toys are literally close to us – in our pockets, on our heads, or on our skin. But they are also close to us in another sense. These gadgets promise to allow us to manage our inner self as it were: our moods, our level of concentration, our sleep, our well-being. They offer new ways to socialize or to express ourselves. They are intimate insofar as they are close to our bodies and relate to our intimate feelings or relationships, but at the same time they intrude upon and interfere with this very intimacy. They promise to allow us to become managers of our own health and happiness – but at the same they may oblige us to move in certain directions or to think about ourselves and our responsibilities in a certain way.

This ostensibly contradictory effect of technology – liberating and constraining at the same time – runs as an important thread through the work of Michel Foucault. In his introduction to *The Use of Pleasure* [47], Foucault explains that his work revolves around three axes of analysis: Knowledge, Power and the Self. In the 1960s, Foucault’s research focussed on (the archaeology of) knowledge, in the 1970s on (the genealogy of) power and in the 1980s on the (ethics of the) self, but in his introduction to *The Use of Pleasure* [47] he emphasises that these three dimensions belong together. In other words, when it comes to assessing extimate gadgets from a critical perspective, three types of questions must be asked: what new forms of knowledge are involved? To which practices of power do they give rise? And finally, which practices of the self are opened up and facilitated by them? In others words, the question is not whether these gadgets are *either* technologies of subjection and control *or* technologies of freedom and emancipation, for the most likely scenario is that they are both. They open up new practices of the self, but *at the same time* and *because of this* they may promote new forms of panoptic surveillance and adaptation, confronting us with societal expectations and standards of normalcy [cf. 48].

In accordance with these three axes of enquiry one could argue first of all that soft enhancement gadgets represent new forms of knowledge, condensed into and represented by high-tech gadgets. This new form of techno-knowledge, however works in two directions. On the one hand it produces enabling devices, allowing individuals to develop and experiment with new practices of the self, exploring new identities, new forms of subjectivity and self-management, closely monitoring psychic and physical parameters. At the same time, these gadgets are technologies of surveillance or control: they keep track of our doings, assess our performance, and literally speak out to us, via messages of various kinds, articulating the imperative that we must change our life in order to live up to societal expectation in terms of health condition, productivity, sociability and level of enjoyment. For indeed: the most pervasive injunction of postmodern neoliberal societies is the injunction to liberate ourselves and to enjoy life to the full; to be happy, productive, innovative, communicative and creative. In other words, although these gadgets come very close, are compatible with an elegant life-style and enter our life-world very smoothly, they may nonetheless be quite intrusive, but in a smooth and subtle way.

Foucault’s contemporary Jacques Lacan likewise explored the profound entanglements of truth, power and the embodied self. Rather than seeing human desire as a rebellious, primitive, natural energy, welling up from below, but thwarted and repressed by power, desire is *produced by power*. Indeed, Lacan stresses the pervasive effects of truth and power on human embodiment, on our “flesh” [49, p. 405]. Moreover, contemporary society actually takes great pains to allow us to thrive and enjoy.^7^ From a Lacanian perspective, the key injunction, coming from the superego, from the powerful Other is: ‘Enjoy!’ [51, p. 320, 52, p. 191; cf. 46]. Seen from this perspective, gadgets actually play a crucial role in the current truth-power-desire constellation, opening up new pathways of desire.

In 1969, for instance, before a crowd of Maoist students, he discussed the famous *Quotations from Chairman Mao Zedong* (the ‘little red book’). Rather than being economically exploited by capitalism, Lacan argued, the working-classes are bereft of a particular type of dexterity or know-how connected with operating big machines, but this type of work is becoming increasingly automated and redundant. Therefore, it struck Lacan that Mao’s booklet still purported to be a ‘manual’ (instructing readers how to manually operate machines). This, he argued, seems outdated. A new type of devices is emerging: very small, functioning smoothly and completely forged by science, known as “gadgets” [53, p. 174]. As an example, Lacan referred to a tiny recording device someone in the audience was actually using to record his speech. As big machines are increasingly being replaced by, or operated with the help of electronic devices (not handled with our hands, but touched by fingertips), traditional industrial know-how is quickly becoming obsolete, while ‘manuals’ inevitably lose their effective potential. It is via these smooth, opaque devices that the voice of power (Φ) now speaks to us. Or as Lacan phrases it three years later (in Seminar XX): science produced a new wave of miniature “gadgets” [54, p. 104] and we have become (in a more radical sense than we are usually aware of) the subjects of these contrivances, which determine the elementary structures of contemporary existence. They connect us with new networks of truth, representing the insatiable desire of the Big Other to acquire more data about human subjects. Go on, continue to produce more data! Never Enough! That is the basic imperative of the new truth-power regime [53, p. 120–121].

Via these gadgets, the gaze of the Other has become distributed and omnipresent, allowing the ‘panoptic Other’ (Foucault), the Lacanian ‘Big Other’ (Lacan), to monitor and survey us, notably while engaged in our most intimate and personalised practices of freedom. By using gadgets, individuals spread clouds of electronic emissions as they move about, feeding the panoptic Other of the terabyte era, who then feeds this information back to them in the form of norms and expectations, highlighting the extent to which they divert from them [48]. It is via these (allegedly hyper-individual) gadgets that the voice of power speaks to us. These gadgets prompt, inform, reprimand and re-educate us. And it is from this perspective that we will analyse the three case studies, presented in the next three sections.

### Case Study 1: Necomimi

Show the world what’s really on your mind and impress your friends with some of the most advanced brainwave technology available! Necomimi’s cat-like reactive movements show how interested or relaxed you are in real-time. It’s a fun, quirky addition to parties, cosplay, bachelorette weekends and tailgating at your favorite sporting event. Anytime you want to entertain your friends and family, wear Necomimi!^8^

Necomimi is a set of cat-ears that users can put on their head. They are connected to an EEG device that measures their attention state. The ear movements reflect their brainwave state. When someone is relaxed, focussed, or something in between, this is reflected by the position of the ears - down, up, or wiggling. As a result, users are promised to become “the centre of attention everywhere you go! People can’t help but watch in fascination as your Necomimi ears move in real-time according to your state of mind.” In commercials, the ears are promoted as a way to express yourself, and to interact with friends. In other words, although Necomimi clearly is a toy, it is also promoted as a socialising tool.

In first instance, Necomimi may seem a joke; something for fun. However, as soon as we start to think it through, and take considerations of participants attending the NERRI workshop on EEG brain-computer interfaces into account, there proves to be more to it than entertainment only. One of the participants for example wondered: “can you imagine that you are in the train and all people are wearing such a thing?” Someone else said: “[think about] job interviews with EEG. When you are too tense, you won’t get the job because you will be susceptible for burn-out.” Also, someone imagined: “What if your boss decides: “from now on, we will measure your brain activity from 9 to 5; this may make you hesitate on Sunday evening: shall I drink this glass of wine, or will this be noticeable in the morning?” What would be the impact of a simple toy like Necomimi if it would really become a hit? How would it affect relationships between people, between strangers on a train, for instance, or between managers and employees at work?

Imagine a train full of people wearing cat ears. What would it mean when an unknown person sitting in front of you keeps wiggling his or her cat ears while staring at you? Or how would it feel if you had a nice conversation with a particular person with cat ears showing interest, but during the chat the ears suddenly go down, suggesting that he or she is bored? Would it add something to ‘naturally occurring’ forms of nonverbal communication? Or imagine having a job interview while wearing cat ears – what would the interviewer think when your ears suddenly slide into the relaxation phase? And how would it be when everyone in the office wears cat ears, and you notice that the ears of your colleagues are continuously alert, while your own ears keep on wiggling? That is to say, even innocent ‘toys’ like Necomimi cat ears may have awkward effects.

Assessing Necomimi in terms of the truth-power-embodied self constellation building on Lacan and Foucault, as outlined above, these uncanny effects should be taken quite seriously. Such gadgets reflect our mood and level of attention. On the one hand they are intimate devices, worn on our bodies, reflecting our emotions, facilitating social interaction, but at the same time they remain something foreign which may easily slip out of our control. It becomes an additional source of information for others (such as colleagues or managers), allowing them to assess our effort and performance. They open us up to the gaze of the Other, who is enabled to monitor and judge us. With a very simple gadget, a new type of knowledge, namely *brainwave knowledge*, is distributed, communicated and interpreted. Necomimi is a social tool, used to amuse friends or colleagues, but to the extent that this gadget becomes embedded and normalised, these friends and colleagues may increasingly expect our brainwaves to live up to the ‘brainwave norm’. Eventually, perhaps, we all will be expected to wiggle too. Indeed, why not? In other words, although Necomimi originates as a self-practice, a form of carefree experimentation, it may actually evolve into a technology of control, giving rise to practices of power, confronting us with new societal expectations and new standards of normalcy. What is so interesting about Necomimi, we would argue, is not the playful device as such, but rather the fact that it represents an emerging generation of communicative technologies bent on making our mood and emotional states more transparent to others, while we ourselves will no longer be totally dependent on verbal and non-verbal communication only when it comes to assessing the state of mind of others. In other words, Necomimi may simply be the first of a new type of communicative gadgets which may enter our daily, social and professional lives.

### Case Study 2: Thync

Thync, how good feels

When you have the power to change the way you feel, it changes everything.

Wear Thync for minutes, feel the effects for hours.

I felt lazy - Now I’m motivated

I felt stressed - Now I’m calm

I felt scattered - Now I’m focused

I felt restless - Now I’m asleep^9^

Thync is a small device, consisting of three components: a strip that connects the right side of your forehead with a spot at the upper side of your neck (to calm down) or at the back of your right ear (to stimulate); a white piece of plastic (a sort of plaster) to put on the strip at your forehead; and an app on your phone with which you can tune the vibes it produces. It combines the technology of tDCS (transcranial direct current stimulation) with TENS (Transcutaneous electrical nerve stimulation) and claims to evoke stimulating or calming vibes. On the Thync website, the device is promoted for purposes such as ‘Get better sleep’, ‘Improve fitness, train harder and recover faster’, ‘Unwind after a stressful day’, ‘Get focused and more productive’, or ‘Be more mindful or centred’.^10^

Thync is a life-style tool. Pictures on the internet show people who are hanging out on the couch or lying in bed, doing sport or yoga or working in the office, having a party or chilling out with friends. It is a new tool (released in June 2015), and the idea is that people can stimulate or calm down with a simple app on their smart phone. Thync is promoted as safe and simple, as is emphasised for example in this text of a press release:

At Home, Work or School, Vibes Enhance Daily Life

Thync Vibes help you smoothly and quickly transition from one mental state to another with comfort and ease. Thync users have described the chemical-free effects to be similar to a “shot of an espresso” or a “glass of wine”. The immediate effects can last from 30 minutes to an hour, with the carry-over impacts lasting several hours.^11^

All this sounds great: Feeling the effects of an espresso or wine whenever you want without experiencing any toxic side-effects. However, controlling your mood with your mobile phone may also be an awkward experience and in the long run it might even fan the flames of the bio-conservative vs. transhumanism debate since it seems to represent the first step towards a process of gradual self-robotisation: (wo) men being increasingly steered by a computer, tuning themselves ‘on’ in the morning and ‘off’ in the evening. Moreover, one may wonder how desirable it really is to be able to adjust one’s mental state. Reducing stress with an app, for example, might make people more resilient in the short term, but perhaps result in burn-out in the longer run.

Moreover, besides as a Foucauldian ‘technology of the self’ [55], Thync may also operate as a technology of surveillance and control. Thync will connect our daily lives with electronic networks. Besides producing knowledge about ourselves (potentially useful in the context of various practices of the self, being enabled to modify our level of activity), others may eventually expect us to relax or concentrate on demand, with the help of a simple smart phone app. Furthermore, such devices may feed the insatiable need of commercial health care companies and governmental agencies for data. They may contribute to the big digital data explosion that is already ongoing, concerning individuals and populations and their preferences, daily routines and social behaviours. That is, the kind of information gathered with the help of simple apps like Thync may feed various ‘Big Others’, who in the end will translate the data collected from consumers into nudging normative expectations. In short, for us, the debate concerning ‘soft’ enhancement gadgets such as Thync should not primarily focus on the question whether there will be any immediate neurological side-effects, risks, or even long-term damage (although this question is highly relevant of course), but should rather focus on the social or cultural impacts, i.e. the ways in which such devices may affect social interactions and expectations: effects which are sometimes referred to as ‘soft’ impacts of technology [56].

### Case Study 3: Alpha-Stim

When I am anxious

When I can’t sleep

When I am depressed

When I am in pain

I Alpha-Stim

Let nothing stop you

In contrast to Necomimi and Thync, Alpha-Stim was originally developed as a therapeutic device. It is a CES (cranial electrotherapy stimulation) device and already exists for more than 20 years. The Alpha-Stim consists of a stimulator and two clips you can put on your earlobes (or electrodes you can put on any other part of your body). To relieve anxiety, depression or sleeping disorders, you may stimulate your brain by sending an electric current from one earlobe to the other. In contrast to psychotherapy, this small electronic device is assumed to work quickly (in the case of depression improvements are expected to set in after 1–3 weeks), or even to give an immediate effect (a reduction in anxiety).

Alpha-Stim is easy to use. It is promoted as a device you can use anywhere and anytime:

Alpha-Stim treatments do not require spending time away from your daily routine. You can use it while you do paperwork, computer work, watching TV or reading the newspaper. (…) You can even stick the device into your pocket and use it while you take a walk, or even during chores.^12^

It is a form of soft enhancement in the sense that it is promoted as safe and easy to use. It is not literally a toy to use for fun, but a relatively simple device to reduce stress, anxiety and depression or to improve sleep. In other words, who would not want an Alpha-Stim to boost their mood at certain moments? Or, as it was expressed by one of the EEG workshop participants: “How about feeling tense or anxious? If you could learn to influence your brainwaves – if I could train myself in such a way that I would never be afraid anymore … that would be cool!” However, another participant immediately formulated an objection in response: “Well, people who don’t feel fear enough: they are the psychopaths.”

That is to say, even with a device that promises to reduce anxieties and depressing feelings, there might be uncanny elements involved. No one wants to be a psychopath, and this reminder illustrates that our fears or moments of depression may also have a function. Just zapping these restraining feelings away with a mere click on a button (so to speak) might also ignore the reasons why someone experiences fear or feels a bit down. From this perspective, the advertisement slogan “Let nothing stop you” suddenly sounds less good. If we are no longer troubled by our fears, our feelings of stress, by depressive thoughts or sleepless nights, something may be lost as well. When there is no longer any reason to stop, when people are always eager to keep going, we may well be worse off than we are now.

In terms of ‘soft’ impacts of ‘soft technologies’ one could argue that, although private use of technologies such as Alpha-Stim for private purposes may seem fairly innocent, they may nonetheless change the way in which we see and think about ourselves. In other words, using Alpha-Stim as a technology of the self may prompt us to rethink our emotional states in terms of brainwave problems [cf. 57]. Fears and inhibitions are reframed as phenomena occurring inside your head, for otherwise it would make no sense to work on your brainwaves. That is, devices such as Alpha-Stim may give rise to neuro-centric understandings of the self. By reducing the ungraspable self to a knowable and refurbishable brain, various options for intra- or interpersonal manipulation are opened up.

### Technologies as Parasites

All three devices discussed, Necomimi, Thync and Alpha-Stim, are extimate devices. They are *in*-timate in the sense that they are wearable close to our body, but also because they allegedly allow us to express or modify inner emotional states. At the same time, they are intrusive and artificial, notably because these technologies may have impacts in the longer run which we initially may not be aware of. The intimacy of Necomimi is obvious – the cat ears say something about you which may otherwise not be shared. It reveals something of what goes on at the inside, on the level of your personal feelings. It makes something public which usually remains intimate. But precisely because of this externalisation of the inner self, there also is a loss of control. Users may become an easier target for manipulative strategies of others. Thync and Alpha-Stim add something to this in the sense that users not only reveal but also may try to influence and control their intimate feelings. But again, this externalisation may eventually imply that others adapt their expectations. That is, all technologies are ‘extimate’ in the sense that they cause part of one’s intimacy (one’s inner state) to become externalised and manipulable.

Necomimi is a social tool in the sense that it is designed to share one’s inner states with others. It is a communicative tool. The wiggling ears literally say something about the user. Thync and Alpha-Stim, on the other hand, allow individuals to adapt to a particular situation. They are adaptive tools, designed for use in daily situations, by individuals who are relating to others (in the office, during yoga class or at a party). The desire to be able to modify one’s inner state indicates that, apparently, we are not always happy with the normal situation. We want to be more in control and to employ soft devices for their mood activating or calming effect. Still, as in the case of Necomini, users give something away about themselves so that they may indicate uncertainties and inhibitions to others (colleagues, managers, partners, etc.).

Moreover, to the extent that the use of devices such as Thync and Alpha-Stim becomes more normalised and less exceptional, even the opposite effect may occur in the sense that others begin to wonder why we do *not* use such device more frequently, so that our moods and inner states become more predictable and manageable. And to the extent that devices such as Alpha-Stim indeed help us to reduce anxieties or depressions easily, we may be criticised for not using it more often whenever our moods or emotions are contrary to what is expected of us. That is, Necomimi, Thync and Alpha-Stim may seem friendly and intimate at first sight, but in the longer run the liberty and autonomy of users may rather decrease than increase.

In other words, these ‘intimate’ devices also involve externalisation, giving other people access to our intimate feelings and desires. And not only particular others (colleagues, managers, teachers, partners), but also the generalised Other, the big panoptic Other, who may now monitor and criticise our performance more intensely than before. These tools may introduce new standards of normalcy, new social expectations. Moreover, we may even gradually become more and more dependent on them. Initially, we may use them for entertainment, or to become more sociable and relaxed, but gradually they will force us to improve our social and professional performance. They may become a kind of externalised super-ego, telling us that we can still do better. Increasingly, we will become dependent on these devices to manage our anxieties and inhibitions.

Extimate technologies are small and smooth and may become easily embedded in our daily lives, but also in our bodies. For although devices such as Alpha-Stim, Thync and Necomimi are not really implanted, they do monitor our inner states and in that sense they become an extended, externalised part of our brains, measuring brain activity and exposing our brain to external influences. Moreover, instead of merely serving us and helping us, these tools may increasingly become objects of daily concern, and as such they may become increasingly demanding: they monitor us and speak to us [35]. They reveal our deficiencies and short-comings, to ourselves, but also to others.

We could think of extimate technologies as devices which open up intimacy to others while inviting otherness into our intimate existence. Thus, extimate technologies may function as technological parasites. We use Thync, Alpha-Stim or Necomimi because we want something: to relax, to feel better, or to improve our social relationships, but before long, the gadget itself becomes the object we are longing for. And instead of solving problems, the gadgets may introduce new concerns into our lives. For instance, because it does not function the way we had hoped it would, or because it works too well: revealing things about ourselves we rather do not want to be informed about, or sharing information about our inner states with others which we do not really want to share. We are increasingly serving the gadgets that were supposed to be serving us.

Extimate technologies are enabling devices. They are designed to make life easier, but at the same time they spur us to change and improve our style of living, subjecting us to an increased self-awareness via processes of self-surveillance. Thus, technologies of the self may easily evolve into technologies of control, subjecting us to the panoptic Other. Or, rather, these devices always involve both dimensions: they are technologies of the self *and* technology of control at the same time. It is precisely *because* we can use these technologies to take better care of ourselves that, in the long run, we may be expected or even *forced* to do so. That is, these extimate technologies are *both* embedded and intrusive; both user-friendly and uncanny.

## Conclusion

The materials produced by the NERRI project reveal that in actual European debates on neuro-enhancement, ‘soft’ enhancement – in the form of neuro-toys or gadgets – may become a focus of attention. In current academic discourse, however, these ‘soft’ devices are underrepresented. By focussing on soft enhancement, the enhancement debate may enter a new stage, bypassing the current tendencies towards either trivialisation or over-dramatization. Instead of asking whether certain types of pills have an effect similar to coffee or whether neuro-tools are transforming humans into cyborgs, ‘soft’ gadgets provide a more interesting target for deliberation. Whenever these technologies are recommended as instances of self-help, or as items that can be used for fun, some of the more questionable aspects seem to be easily overlooked. Something more is involved than mere entertainment or relaxation and this article aimed to articulate this ‘more’.

Soft enhancement technologies are basically gadgets individuals may want to use to express themselves or to take care of themselves. But this objective – expressing and sharing one’s inner feelings or changing one’s inner state – may also subject users to surveillance or even manipulation by others. And although this surveillance or manipulation argument might seem to fit the agenda of bio-conservatives, we hope to have made clear that we are not referring to dystopian scenarios of thought police or oppression, but to a more subtle form of power as indicated by Foucault and Lacan. These technologies are designed to enhance our lives, but can also become an issue of concern. These gadgets are extimate rather than intimate because their intimacy exposes us to the gaze of others. They are both intimate and intrusive, both familiar and uncanny. We could even think of these neuro-toys as technological parasites: using *us* to function and proliferate.

## Footnotes

1 This idea to think about technologies as parasites was inspired by J.A. Miller in his text on Intimity (2008), in which he writes: “Extimacy says that the intimate is Other-like a foreign body, a parasite.” http://www.lacan.com/symptom/?p=36

2
http://www.nerri.eu/eng/home.aspx

3
*Ciência Viva*, Portugal; *London School of Economics and Political Science*, United Kingdom; *Radboud University Nijmegen*, the Netherlands; *Austrian Academy of Sciences*, Austria; *Scuola Internazionale Superiore di Studi Avanzati*, Italy; *Instituto de Biologia Molecular e Celular*, Portugal; *Experimentarium*, Denmark; *Tilburg Law School*, the Netherlands; *Central European University*, Hungary; *Universität Stuttgart*, Germany*; Johannes Gutenberg Universität* Mainz, Germany; *Johannes Kepler Universität*, Austria; *Universitat Pompeu Fabra*, Spain; *Toscana Life Sciences*, Italy; *University of Iceland*, Iceland; *Genetic Alliance*, United Kingdom; *European Brain Council*, Belgium; *King’s College London*, United Kingdom.

4 For this paper we analysed all general reports (see: http://www.nerri.eu/eng/mutual-learning-exercises.aspx), and the notes and transcripts of those events we had access to because we were (directly or indirectly) involved in the organization (MLE 1,3,38,39,44,50,55) or that were made available on the internet (MLE 12, https://www.youtube.com/watch?v=Eioy_sIICiU)

5 MLE 39 see http://www.nerri.eu/eng/mutual-learning-exercises/mle-39-world-cafe-enschede-tdcs.aspx

6 MLE 38 see http://www.nerri.eu/eng/mutual-learning-exercises/mle-38-world-caf%C3%A9-enschede-eeg.aspx

7 When asked whether (contrary to traditional psychoanalytic views, criticized by him) Lacan’s work represented a view much closer to his own, Foucault replied: “No comment, as politicians say when presented with a question which embarrasses them” [50, p. 666].

8 See: http://www.necomimi.com/ [accessed December 2015]. This case was presented during MLE 38, see http://www.nerri.eu/eng/mutual-learning-exercises.aspx)

9 See http://www.thync.com/archive/home, accessed in November 2015

10 The THYNC device is tested, but only by its own developers. The FDA considered the THYNC system as a life style product and hence not ‘subject to medical device regulations requiring pre-market clearance or approval’. (http://support.thync.com/articles/FAQ/H08)

11
http://www.thync.com/resources/press-release/thync-launches-first-wearable-to-shift-your-state-of-mind

12
https://www.jamesgfriesen.com/alpha-stim.php
